# Design, Synthesis and Antifibrotic Activities of Carbohydrate- Modified 1-(Substituted aryl)-5-trifluoromethyl-2(1*H*) Pyridones

**DOI:** 10.3390/molecules17010884

**Published:** 2012-01-17

**Authors:** Qinghua Lou, Xiangbao Meng, Zhiqi Lao, Lingling Xuan, Jinye Bai, Qi Hou, Gaoyun Hu, Renna Luo, Lijian Tao, Zhongjun Li

**Affiliations:** 1 State Key Laboratory of Natural and Biomimetic Drug, Department of Chemical Biology, School of Pharmaceutical Sciences, Peking University, Beijing 100191, China; 2 Department of Pharmacology, Institute of Materia Medica, Chinese Academy of Medical Sciences and Peking Union Medical College, Beijing 100050, China; 3 Department of Medicinal Chemistry, School of Pharmaceutical Sciences, Central South University, Changsha, Hunan 410013, China; 4 Division of Nephrology, Xiangya Hospital, Central South University, Changsha, Hunan 410008, China

**Keywords:** antifibrotic, pirfenidone, fluorofenidone, carbohydrate modified, glucose

## Abstract

Pirfenidone, a pyridone compound, is an effective and novel antifibrotic agent. In this article, we describe the design, synthesis and activity evaluation of novel antifibrotic agents, 1-(substituted aryl)-5-trifluoromethyl-2(1*H*) pyridones modified with carbohydrate. Most of the title compounds exhibited comparable or better inhibitory activity than fluorofenidone. Notably, compound **19a** demonstrated the highest cell-based inhibitory activity against NIH 3T3 (IC_50_ = 0.17 mM).

## 1. Introduction

Fibrosis of organs and tissues (e.g., heart, lungs, liver, kidneys, blood vessel and skin) is an important pathological change associated with many diseases that are major causes of human morbidity and mortality [1,2]. Generally, fibrosis can occur as a consequence of different pathological conditions, such as tissue damage, inflammatory diseases, foreign implants, and tumors. It is clear that fibrosis of different organs and tissues possesses the same feature of fibroblast accumulation and excess deposition of extracellular matrix (ECM), which leads to distorted organ architecture and function [3,4]. There are various cytokines, chemokines, and growth factors involved in the development of fibrosis [5]. In spite of the increased knowledge about the pathogenesis of fibrosis and the different mediators involved, there is still no effective treatment for fibrosis.

**Figure 1:**
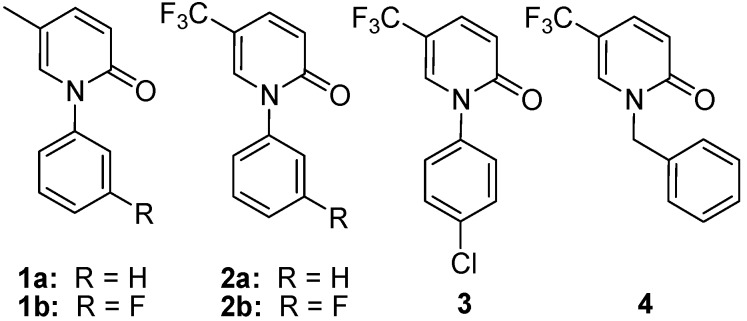
Structures of Pirfenidone and analogues.

Pirfenidone (5-methyl-1-phenyl-2(1*H*)-pyridone, Figure 1, compound **1a**), is an effective and novel antifibrotic agent that is potentially effective for the treatment of idiopathic pulmonary fibrosis (IPF) [6] that was launched in Japan in 2008. Pirfenidone can inhibit fibroblast proliferation and collagen synthesis, and it ameliorates bleomycin-induced and cyclophosphamide-induced lung fibrosis [7,8]. In order to improve the antifibrotic activity of pirfenidone, a series of pirfenidone derivatives (Figure 1) were synthesized [9,10,11,12]. Fluorofenidone (Figure 1, compound **1b**), an analogue of pirfenidone, shows equivalent antifibrotic activity, lower toxicity and longer half-life than the parent compound pirfenidone. However, the activities of pirfenidone and fluorofenidone are relatively weak; their effective doses are 500 mg/kg for the treatment of renal fibrosis in rats and the regular dose of pirfenidone for Japanese patients with IPF is 400–600 mg three-times daily [6,13]. The metabolized products have no *in vivo* activity, which could be the cause of high effective doses of pirfenidone and fluorofenidone. Replacement of the methyl group with trifluoromethyl (Figure 1) could protect the derivative from metabolism, but trifluoromethyl substitution increases lipid solubility and toxicity. Therefore, it is necessary to prepare more effective antifibrotic agents with novel chemical structures and improved water solubility.

The aim of this study was to prepare antifibrotic compounds with higher water solubility. Because carbohydrates are a kind of polyhydroxy compounds with no toxicity and high water solubility, attachment of a carbohydrate moiety should increase hydrophilicity [14]. The modification with carbohydrate also influences the pharmacokinetic properties of the modified compounds. Recent advancements in molecular glycobiology have facilitated the development of effective glycodrugs [15]. A series of *N*-substituted 1-(4-amino-2-chlorophenyl)-5-trifluoromethyl-2(1*H*) pyridones were previously synthesized and tested for NIH 3T3 inhibitory activity [10]. Therefore, we selected amines **15–19** (Table 1, R = H) as parent compounds for modification with monosaccharides. Herein, we report the design, synthesis and biological evaluation of carbohydrate-modified 1-(substituted aryl)-5-trifluoromethyl-2(1*H*) pyridones. The structures of the final target compounds are shown in Table 1. We explored the SAR using methyl 6-deoxy-α-D-glucopyranoside, methyl 6-deoxy-α-D-mannopyranoside and methyl 6-deoxy-β-D-galactofuranoside.

**Table 1:** NIH 3T3 inhibitory activity. 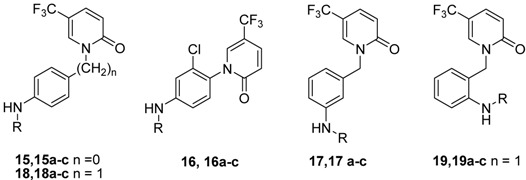

Compound	R	IC_50_ (mM) *	Compound	R	IC_50_ (mM) *
**1b**	--	4.18	**18**	H	1.31
**15a**	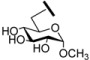	3.93	**18a**	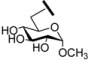	2.47
**15b**	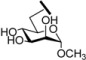	5.68	**18b**	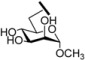	13.17
**15c**	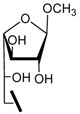	8.40	**18c**	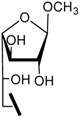	3.30
**16**	H	0.46	**19**	H	1.71
**16a**	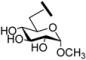	N.D. ^a^	**19a**	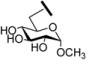	0.17
**16b**	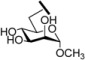	1.13	**19b**	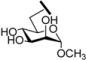	0.79
**16c**	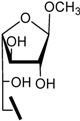	N.D. ^a^	**19c**	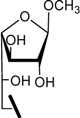	0.84
**17a**	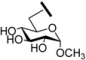	2.63			
**17b**	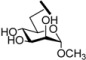	5.81			
**17c**	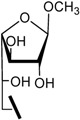	0.42			

* *p* < 0.05; ^a^ N.D.: not determined.

## 2. Results and Discussion

**Scheme 1:**
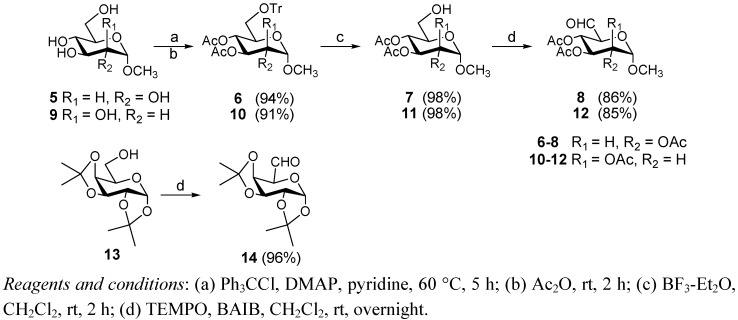
Synthesis of compounds **8**, **12** and **14**.

The preparation of methyl 2,3,4-tri-*O*-acetyl-6-aldehydo-α-D-gluco-hexodialdo-1,5-pyranoside (**8**) and methyl 2,3,4-tri-*O*-acetyl-6-aldehydo-α-D-manno-hexodialdo-1,5-pyranoside (**12**) is described in Scheme 1. Methyl 2,3,4-tri-*O*-acetyl-6-*O*-trityl-α-D-glucopyranoside (**6**) was prepared directly from methyl α-D-glucopyranoside in two steps. Removal of trityl protection was accomplished by treatment with boron trifluoride etherate in methanol and methylene chloride to produce compound **7**[16]. Subsequently selective oxidation of a primary hydroxyl group to an aldehydo group was performed at room temperature with a mild oxidation reagent, 2,2,6,6-tetramethyl-1-piperidinyloxy (TEMPO) and [bis(acetoxy)iodo]benzene (BAIB) [17]. Using the same method, compound **12** was obtained from methyl α-D-mannopyranoside **9**. Galactose aldehyde can be prepared with a isopropylidene moiety as a protective group (Scheme 1). Isopropylidenation of D-galactose with sulfuric acid-copper sulfate as catalyst in acetone produced 1,2;3,4-di-*O*-isopropylidene-D-galactose (**13**) in 58.3% yield [18]. The conversion of compound **13** to **14** was performed with the same method used in the conversion of compound **7** to **8**. The structure of compounds **7–8** and **11–14** were in accordance with their reported spectroscopic data [19,20,21]. 

**Scheme 2:**
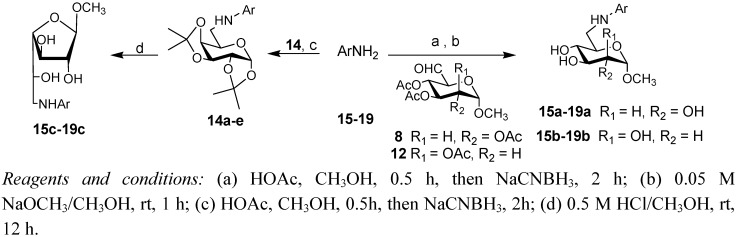
Synthesis of compounds **15a-19a**, **15b-19b** and **15c-19c**.

The synthesis of **15a-19a** and **15b-19b** is shown in Scheme 2. Nucleophilic addition of the aldehyde **8** or **12** with amine **15–19** [10] in methanol and acetic acid followed by dehydration produced the corresponding imines, which were further reduced with sodium cyanoborohydride to give the corresponding secondary amines [22]. Amines were converted to the desired compounds **15a-19a** and **15b–19b** by treatment with sodium methoxide in methanol. The synthesis of **15c–19c** is also described in Scheme 2. Using the same method [22], secondary amines **14a–e** were prepared. Treatment of **14a–e** with a solution of 0.5 M HCl/CH_3_OH under room temperature, caused removal of the isopropylidene groups and the formation of methyl β-D-galactofuranosides **15c–19c** as the main product. Under these conditions, deprotection of **14a** also gave a minor amount of the corresponding galactopyranoside (5.7%) except for **15c**, but deprotection of **14b–e** gave little of the corresponding galactopyranosides (<1%).

### Biological Assay

A cell-based fibrosis inhibition assay was performed to evaluate the inhibitory effects of the prepared compounds on NIH 3T3 cells. NIH 3T3 (mouse embryonic lung fibroblast, State Key Laboratory of Genetics) cells were seeded on 96-well plates (Costar) in DMEM containing 10% NBS (3 × 10^4^ cell per well in 100 μL medium) in humidified air with 5% CO_2_ at 37 °C. Different concentrations of test compounds and fluorofenidone were added to the medium, and DMSO was used as a solvent for the test compounds and fluorofenidone, which were applied at a final concentration of 0.1% (v/v) in cell culture medium. Cells were then incubated for 48 hours, followed by detecting with MTT spectrophotometry. Briefly, 5 mg/mL MTT (100 μL) was added to each well, and incubated for 4 hours at 37 °C. All liquid in each well was then discarded and DMSO (150 μL) was added. The OD at 570 nm was detected by spectrometry. IC_50_ was determined by nonlinear regression analysis using GraphPad PRISM.

Of the aryl amines connected with a carbohydrate that were tested, compound **19a** exhibited the highest inhibitory activity against NIH 3T3 (IC_50_ = 0.17 mM) among the five aryl amines modified with glucose. When the carbohydrate moiety was mannose, **19b** appeared to have the highest inhibitory activity (IC_50_ = 0.79 mM). When the carbohydrate moiety was galactose, **17c** demonstrated the highest inhibitory activity (IC_50_ = 0.42 mM), while **19c** showed two-fold lower inhibitory activity (IC_50_ = 0.84 mM) than **17c**. Thus, among the five aryl amines that were modified with carbohydrate, **19** yielded more active compounds than the other aryl amines.

The same aryl amine-type compounds were also explored. Compound **15a** demonstrated better inhibitory activity than **15b** and **15c** (IC_50_ = 3.93 mM for **15a**, IC_50_ = 5.68 mM for **15b**, IC_50_ = 8.40 mM for **15c**); compound **17c** showed the highest inhibitory activity among compounds **17a–c** (IC_50_ = 2.63 mM for **17a**, IC_50_ = 5.81 mM for **17b**, IC_50_ = 0.42 mM for **17c**); compound **18a** appeared to have significantly higher activity than **18b** and slightly better activity than **18c** in NIH 3T3 inhibition (IC_50_ = 2.47 mM for **18a**, IC_50_ = 13.17 mM for **18b**, IC_50_ = 3.30 mM for **18c**); compound **19a** exhibited better activity than either **19b **or **19c** (IC_50_ = 0.17 mM for **19a**, IC_50_ = 0.79 mM for **19b**, IC_50_ = 0.84 mM for **19c**). These results indicate that among the three carbohydrates used for the modification of pirfenidone analogues, glucose appeared to be a better candidate (e.g., **19a**, IC_50_ = 0.17 mM), although some compounds that were modified with galactose also had high inhibitory activity (e.g., **17c**, IC_50_ = 0.42 mM).

Compounds **17**, **18**, and **19** differ in the NHR (R= various carbohydrate moieties) positions. When NHR was modified at the *ortho* position, the antifibrotic activities of all three compounds were increased, especially compound **19a** which showed ten-fold higher activity compared with compound **19** (R = H); when NHR was modified at the *para* position, the antifibrotic activities were decreased; when NHR was modified at the *meta* position, compound **17c** showed better antifibrotic activities compared with **17a** and **17b**. Among these compounds, compound **19a**, that is NHR modified with glucose at the *ortho* position, exhibited the highest antifibrotic activity.

When a CH_2_ group was used to link the substituted phenyl ring with 5-trifluoromethyl-2(1*H*)-pyridone (Table 1, compounds **18a–c** and **19a–c**), some of the resulting products showed obviously higher NIH 3T3 inhibitory activities (Table 1, compound **19a–c**), perhaps due to the increased flexibility between two substituted aryl rings.

Some modifications of pirfenidone analogues with different monosaccharides appeared to increase the inhibitory activity. For example, compounds **19a–c**, which were prepared by modification of pirfenidone analogue **19** with a carbohydrate ring, showed a significant increase of inhibitory activity in NIH 3T3 cells. Compound **19a** demonstrated ten-fold higher activity compared with pirfenidone analogue **19**, but modifications of **16** and **18** with carbohydrates led to decreases in inhibitory activities against NIH 3T3 cells.

## 3. Experimental

### 3.1. General

Unless specified, all reagents and starting materials were purchased from commercial sources and used as received. Solvents were purified following standard literature procedures. Analytical TLC was performed on silica gel60 F_254_ precoated on glass plates, with detection by fluorescence and/or by staining with 5% concentrated sulphuric acid in EtOH. ^1^H- (400 MHz) and ^13^C-NMR spectra (100 MHz) were recorded on a Bruker DRX-400 spectrometer at 25 °C. Chemical shifts *(δ)* for ^1^H and ^13^C spectra are expressed in ppm relative to internal Me_4_Si as standard. Signals were abbreviated as s, singlet; bs, broad singlet; d, doublet; t, triplet; q, quartet; m, multiplet. Sugar signals were numbered as customary. ESI mass spectra and high resolution mass spectrometry (HRMS) were recorded using an Agilent Technologies 1100 Series instrument (ESI ionization). Optical rotations were measured in a 1.00 dm tube with an Optical Activity AA-10R polarimeter in methanol or chloroform.

### 3.2. General Procedure for the Synthesis of Compounds **14a–e**

Amine (compounds **15–19**, 0.5 mmol) was added to a solution of compounds **14** (155 mg, 0.6 mmol) in dry methanol (2 mL) and acetic acid (1.5 mL) under stirring and under a nitrogen atmosphere at room temperature. After 10 min NaCNBH_3_ (40 mg, 0.6 mmol) was added. The solution was stirred at room temperature for 30 min. After completion of the reaction (TLC 3:1 petroleum ether b.p. 60–90 °C-EtOAc), the methanol was evaporated under reduced pressure, the reaction mixture was diluted with CH_2_Cl_2_ (5 mL) and washed with saturated NaHCO_3_ (3 × 5 mL) and with water (5 mL). The organic phase was dried over sodium sulphate and the solvent was evaporated under reduced pressure to give a crude mass which was purified by flash chromatography (4:1 petroleum ether b.p. 60–90 °C-EtOAc). 

*1,2;3,4-di-O-Isopropylidene-6-deoxy-6-(4-(5-trifluoromethyl-2(1*H*)-pyridone-1-yl)-anilino)-α-D-galactopyranose* (**14a**). Compound **15** (127 mg, 0.5 mmol) was used to prepare compound **14a** (205 mg, 82.3%) as a colorless syrup; [α]^25^_D_ −75 (c 0.10, CHCl_3_); ^1^H-NMR (CDCl_3_): δ 7.72 (s, 1H), 7.46 (dd, *J*_1_ = 2.8 Hz, *J*_2_ = 9.6 Hz, 1H), 7.15–7.13 (m, 2H), 6.73–6.68 (m, 3H), 5.56 (d, *J* = 5.2 Hz, 1H, H-1), 4.64 (dd, *J*_1_ = 2.4 Hz, *J*_2_ = 8.0 Hz, 1H, H-3), 4.34 (dd, *J*_1_ = 2.4 Hz, *J*_2_ = 5.2 Hz, 1H, H-2), 4.26 (dd, *J*_1_ = 2.0 Hz, *J*_2_ = 8.0 Hz, 1H, H-4), 4.04–4.00 (m, 1H, H-5), 3.40–3.37 (m, 2H, H-6), 1.48 (s, 3H, CH_3_), 1.44 (s, 3H, CH_3_), 1.37 (s, 3H, CH_3_), 1.33 (s, 3H, CH_3_); ^13^C-NMR(CDCl_3_): δ 162.1 (C=O), 148.7, 138.3, 138.2, 135.0, 129.7, 122.0, 113.2, 109.5, 109.1, 108.7, 96.4 (C-1), 71.6, 70.8, 70.6 and 65.7 (C-2, C-3, C-4, C-5), 43.9 (C-6), 26.0 (CH_3_), 26.0 (CH_3_), 24.9 (CH_3_), 24.4 (CH_3_); ESI-MS: Calcd for C_24_H_27_N_2_O_6_F_3_: 497.2[M+H]^+^, found: 497.2[M+H]^+^.

*1,2;3,4-di-O-Isopropylidene-6-deoxy-6-(3-chloro-4-(5-trifluoromethyl-2(1*H*)-pyridone-1-yl)-anilino)-α-D-galactopyranose* (**14b**). Compound **16** (145 mg, 0.5 mmol) was used to prepare compound **14b** (195 mg, 73.6%) as a colorless syrup; [α]^25^_D_ −59 (c 0.10, CHCl_3_); ^1^H-NMR (CDCl_3_): δ 7.58 (s, 1H), 7.51 (dd, *J*_1_ = 2.8 Hz, *J*_2_ = 9.6 Hz, 1H), 7.10 (dd, *J*_1_ = 1.6 Hz, *J*_2_ = 8.8 Hz, 1H), 6.78 (d, *J* = 2.4 Hz, 1H), 6.71 (d, *J* = 2.8 Hz, 1H), 6.63–6.60 (m, 1H), 5.56 (d, *J* = 5.2 Hz, 1H, H-1), 4.64 (dd, *J*_1_ = 2.4 Hz, *J*_2_ = 8.0 Hz, 1H, H-3), 4.35 (dd, *J*_1_ = 2.4 Hz, *J*_2_ = 5.2 Hz, 1H, H-2), 4.26 (dd, *J*_1_ = 2.0 Hz, *J*_2_ = 8.0 Hz, 1H, H-4), 4.02–3.99 (m, 1H, H-5), 3.38–3.35 (m, 2H, H-6), 1.48 (s, 3H, CH_3_), 1.47 (s, 3H, CH_3_), 1.37 (s, 3H, CH_3_), 1.34 (s, 3H, CH_3_); ^13^C-NMR (CDCl_3_): δ 161.7 (C=O), 150.0, 138.7, 135.5, 132.0, 129.2, 126.5, 122.3, 113.3, 112.2, 112.1, 109.6, 108.8, 96.4 (C-1), 71.6, 70.8, 70.6 and 65.6 (C-2, C-3, C-4, C-5), 43.8 (C-6), 26.0 (CH_3_), 24.9 (CH_3_), 24.4 (CH_3_); ESI-MS: Calcd for C_24_H_26_N_2_O_6_F_3_Cl: 531.2 [M+H]^+^, found: 531.1 [M+H]^+^.

*1,2;3,4-di-O-Isopropylidene-6-deoxy-6-(3-(5-trifluoromethyl-2(1*H*)-pyridone-1-yl-methylene)-anilino)-α-D-galactopyranose* (**14c**). Compound **17** (135 mg, 0.5 mmol) was used to prepare compound **14c**(192 mg, 75.3%) as a colorless syrup; [α]^25^_D_ −47 (c 0.10, CHCl_3_); ^1^H-NMR (CDCl_3_): δ 7.63 (s, 1H), 7.41 (d, *J* = 1.6 Hz, 1H), 7.18–7.14 (m, 1H), 6.67–6.59 (m, 4H), 5.54 (d, *J* = 4.8 Hz, 1H, H-1), 5.04 (m, 2H, CH_2_), 4.62 (dd, *J*_1_ = 1.6 Hz, *J*_2_ = 8.0 Hz, 1H, H-3), 4.33 (t, *J* = 2.4 Hz, 1H, H-2), 4.25 (d, *J* = 7.6 Hz, 1H, H-4), 3.98 (d, *J* = 4.8 Hz, 1H, H-5), 3.40–3.29 (m, 2H, H-6), 1.47 (s, 3H, CH_3_), 1.37 (s, 3H, CH_3_), 1.36 (s, 3H, CH_3_), 1.31 (s, 3H, CH_3_); ^13^C-NMR (CDCl_3_): δ 161.9 (C=O), 148.8, 136.7, 136.1, 134.8, 130.0, 121.5, 117.3, 113.1, 109.5, 108.7, 96.4 (C-1), 71.7, 70.8, 70.6 and 65.7 (C-2, C-3, C-4, C-5), 52.3 (CH_2_), 43.9 (C-6), 26.0 (CH_3_), 25.8 (CH_3_), 24.9 (CH_3_), 24.4 (CH_3_); ESI-MS: Calcd for C_25_H_29_N_2_O_6_F_3_: 511.2 [M+H]^+^, found: 511.2 [M+H]^+^.

*1,2;3,4-di-O-Isoprop*y*lidene-6-*deoxy*-6-(4-(5-trifluoromethyl-2(1*H*)-pyridone-1-yl-methylene)-anilino)-α-D-galactopyranose* (**14d**). Compound **18** (135 mg, 0.5 mmol) was used to prepare compound **14d **(207 mg, 81.2%) as a colorless syrup; [α]^25^_D_ −68 (c 0.10, CHCl_3_); ^1^H-NMR (CDCl_3_): δ 7.60 (s, 1H), 7.39 (dd, *J*_1_ = 2.0 Hz, *J*_2_ = 9.6 Hz, 1H), 7.15 (d, *J* = 8.4 Hz, 2H), 6.65–6.62 (m, 3H), 5.54 (d, *J*= 4.8 Hz, 1H, H-1), 5.00 (s, 2H, CH_2_), 4.62 (dd, *J*_1_ = 2.0 Hz, *J*_2_ = 8.0 Hz, 1H, H-3), 4.33–4.31 (m, 1H, H-2), 4.25–4.23 (m, 1H, H-4), 3.99 (t, *J* = 6.0 Hz, 1H, H-5), 3.41–3.30 (m, 2H, H-6), 1.47 (s, 3H, CH_3_), 1.36 (s, 3H, CH_3_), 1.33 (s, 3H, CH_3_), 1.30 (s, 3H, CH_3_); ^13^C-NMR (CDCl_3_): δ 162.0 (C=O), 148.4, 136.4, 136.3, 134.7, 134.6, 130.1, 123.5, 121.4, 113.6, 109.5, 108.7, 96.4 (C-1), 71.7, 70.8, 70.6 and 65.6 (C-2, C-3, C-4, C-5), 52.1 (CH_2_), 43.9 (C-6), 26.0 (CH_3_), 25.7 (CH_3_), 24.9 (CH_3_), 24.4 (CH_3_); ESI-MS: Calcd for C_25_H_29_N_2_O_6_F_3_: 511.2 [M+H]^+^, found: 511.2 [M+H]^+^.

*1,2;3,4-di-O-Isopropylidene-6-deoxy-6-(2-(5-trifluoromethyl-2(1*H*)-pyridone-1-yl-methylene)-anilino)-α-D-galactopyranose* (**14e**). Compound **19** (135 mg, 0.5 mmol) was used to prepare compound **14e** (210 mg, 82.4%) as a colorless syrup; [α]^25^_D_ −62 (c 0.10, CHCl_3_); ^1^H-NMR (CDCl_3_): δ 7.68 (s, 1H), 7.42 (dd, *J*_1_ = 2.0 Hz, *J*_2_ = 9.6 Hz, 1H), 7.25 (m, 1H), 7.14 (d, *J* = 7.2 Hz, 1H), 6.72–6.64 (m, 3H), 5.44 (d, *J* = 5.2 Hz, 1H, H-1), 5.20–4.95 (m, 2H, CH_2_), 4.57 (dd, *J*_1_ = 2.0 Hz, *J*_2_ = 8.0 Hz, 1H H-3), 4.27 (dd, *J*_1_ = 2.0 Hz, *J*_2_ = 5.2 Hz, 1H H-2), 4.21 (d, *J* = 8.8 Hz, 1H, H-4), 3.94 (t, *J* = 6.4 Hz, 1H, H-5), 3.50–3.43 (m, 1H, H-6), 3.36–3.31 (m, 1H, H-6), 1.47 (s, 3H, CH_3_), 1.34 (s, 3H, CH_3_), 1.26 (d, 6H, 2xCH_3_); ^13^C-NMR (CDCl_3_): δ 162.4 (C=O), 146.6, 136.1, 136.1, 135.0, 131.3, 130.5, 121.3, 119.3, 116.9, 111.6, 110.3, 109.3, 108.5, 96.4 (C-1), 71.4, 70.8, 70.6 and 65.1 (C-2, C-3, C-4, C-5), 49.1 (CH_2_), 43.6 (C-6), 26.0 (CH_3_), 25.6 (CH_3_), 24.9 (CH_3_), 24.4 (CH_3_); ESI-MS: Calcd for C_25_H_29_N_2_O_6_F_3_: 511.2 [M+H]^+^, found: 511.2 [M+H]^+^.

### 3.3. General Procedure for the Synthesis of Compounds **15a–19a** and **15b–19b**

Amine (compound **15–19**, 0.5 mmol) was added to a solution of compounds **8** or **12** (0.6 mmol) in dry methanol (2 ml) and acetic acid (1.5 mL) under stirring and under nitrogen atmosphere at room temperature. After 10 min NaCNBH_3_ (40 mg, 0.6 mmol) was added. The solution was stirred at room temperature for 30 min. After completion of the reaction (TLC 3:1 petroleum ether b.p. 60–90 °C-EtOAc), the methanol was evaporated under reduced pressure, the reaction mixture was diluted with CH_2_Cl_2_ (5 mL) and washed with saturated NaHCO_3 _(3 × 5 mL) and with water (5 mL). The organic phase was dried over sodium sulphate and the solvent was evaporated under reduced pressure to give a crude mass which was dissolved in 0.05 M NaOMe/MeOH (5 mL) and stirred at ambient temperature for 30 min. The stirring reaction mixture was neutralized with prewashed Amberlite IR 120-H^+^. The resin was filtered off and the solvent was evaporated under reduced pressure. The crude product was purified by flash chromatography (7:1 CHCl_3_-MeOH).

*Methyl 6-deoxy-6-(4-(5-trifluoromethyl-2(1*H*)-pyridone-1-yl)-anilino)-α-D-glucopyranoside* (**15a**). Compound **15** (127 mg, 0.5 mmol) and **8** (190 mg, 0.6 mmol) were used to prepare compound **15a** (143 mg, 66.5%) as a colorless syrup; [α]^25^_D_ −63 (c 0.10, MeOH); ^1^H-NMR (MeOH-*d*_4_) δ 7.97 (s, 1H), 7.66 (dd, *J*_1_ = 2.4 Hz, *J*_2_ = 9.6 Hz, 1H), 7.01 (d, *J* = 8.8 Hz, 2H), 6.76 (d, *J* = 8.4 Hz, 2H), 6.64 (d, *J* = 9.2 Hz, 1H), 4.62 (d, *J* = 3.6 Hz, 1H, H-1), 3.71–3.63 (m, 1H), 3.59–3.55 (m, 2H), 3.38–3.35 (m, 1H), 3.28 (s, 3H), 3.22–3.17 (m, 2H); ^13^C-NMR (MeOH-*d*_4_) δ 164.5 (C=O), 151.0, 140.6, 140.5, 137.3, 130.3, 128.1, 122.2, 113.6, 101.2 (C-1), 75.1, 73.6, 73.5 and 71.4 (C-2, C-3, C-4, C-5), 55.5 (OCH_3_), 45.7 (C-6); HRMS: Calcd for C_19_H_21_N_2_O_6_F_3_ [M+H]^+^ 431.1431, found 431.1425.

*Methyl* *6-deoxy-6-(3-chloro-4-(5-trifluoromethyl-2(1*H*)-pyridone-1-yl)-anilino)-α-D-glucopyranoside* (**16a**). Compound **16** (145 mg, 0.5 mmol) and **8** (190 mg, 0.6 mmol) were used to prepare compound **16a** (152 mg, 65.5%) as a colorless syrup; [α]^25^_D_ −34 (c 0.10, MeOH); ^1^H-NMR (MeOH-d_4_): δ 7.92 (s, 1H), 7.70 (dd, *J*_1_ = 2.8 Hz, *J*_2_ = 9.6 Hz, 1H), 7.08 (d, *J* = 8.8 Hz, 2H), 6.84 (m, 1H), 6.68 (m, 2H), 4.62 (d, *J* = 3.6 Hz, 1H, H-1), 3.63–3.60 (m, 1H), 3.59–3.51 (m, 2H), 3.38–3.34 (m, 1H), 3.29 (s, 3H, OCH_3_), 3.26–3.17 (m, 3H); ^13^C-NMR (MeOH-d_4_): δ 164.1 (C=O), 152.5, 141.2, 137.8, 132.7, 130.2, 126.7, 122.5, 113.8, 112.8, 101.2 (C-1), 75.1, 73.6, 73.3 and 71.6 (C-2, C-3, C-4, C-5), 55.6 (OCH_3_), 45.5 (C-6); HRMS: Calcd for C_19_H_21_N_2_O_6_F_3_Cl: 465.1041 [M+H]^+^, found: 465.1038 [M+H]^+^.

*Methyl* *6-deoxy-6-(3-(5-trifluoromethyl-2(1*H*)-pyridone-1-yl-methylene)-anilino)-α-D-glucopyranoside* (**17a**). Compound **17** (135 mg, 0.5 mmol) and **8** (190 mg, 0.6 mmol) were used to prepare compound **17a** (150 mg, 67.6%) as a colorless syrup; [α]^25^_D_ −45 (c 0.10, MeOH); ^1^H-NMR (MeOH-d_4_): δ 8.06 (s, 1H), 7.55 (dd, *J*_1_ = 2.4 Hz, *J*_2_ = 9.6 Hz, 1H), 7.02 (t, *J* = 8.0 Hz, 1H), 6.61–6.58 (m, 3H), 6.49 (d, *J* = 7.6 Hz, 1H), 5.03 (s, 2H, CH_2_), 4.57 (d, *J* = 2.8 Hz, 1H, H-1), 3.62–3.48 (m, 3H), 3.35–3.12 (m, 1H), 3.16 (s, 3H, OCH_3_), 3.19–3.06 (m, 2H); ^13^C-NMR (MeOH-d_4_): δ 161.2 (C=O), 147.8, 136.6, 136.6, 135.0, 134.1, 127.8, 123.3, 120.6, 118.9, 114.6, 111.3, 111.0, 108.7, 108.4, 98.2 (C-1), 72.2, 70.7, 70.7 and 68.19 (C-2, C-3, C-4, C-5), 52.5 (OCH_3_), 50.9 (CH_2_), 43.0 (C-6); HRMS: Calcd for C_20_H_23_N_2_O_6_F_3_: 445.1587 [M+H]^+^, found: 445.1583 [M+H]^+^.

*Methyl 6-deoxy-6-(4-(5-trifluoromethyl-2(1H)-pyridone-1-yl-methylene)-anilino)-α-D-glucopyranoside* (**18a**). Compound **18** (135 mg, 0.5 mmol) and **8** (190 mg, 0.6 mmol) were used to prepare compound **18a** (155 mg, 69.8%) as a colorless syrup; [α]^25^_D_ −54 (c 0.10, MeOH); ^1^H-NMR (MeOH-d_4_): δ 8.03 (s, 1H), 7.54 (dd, *J*_1_ = 2.8 Hz, *J*_2_ =9.6 Hz, 1H), 7.07 (d, *J* = 8.4 Hz, 2H), 6.64 (d, *J* = 4.4 Hz, 2H), 6.56 (d, *J* = 9.6 Hz, 1H), 4.98 (s, 2H, CH_2_), 4.57 (d, *J* = 3.6 Hz, 1H, H-1), 3.63–3.58 (m, 1H), 3.55–3.49 (m, 2H), 3.34–3.31 (m, 1H), 3.24–3.23 (m, 1H), 3.18 (s, 3H, OCH_3_), 3.15–3.06 (m, 2H); ^13^C-NMR (MeOH-d_4_): δ 162.8 (C=O), 148.9, 137.7, 137.7, 135.5, 129.3, 123.5, 120.3, 113.1, 99.8 (C-1), 73.7, 72.2, 72.2 and 69.6 (C-2, C-3, C-4, C-5), 54.0 (OCH_3_), 52.1 (CH_2_), 44.5 (C-6); HRMS: Calcd for C_20_H_23_N_2_O_6_F_3_: 467.1400 [M+Na]^+^, found: 465.1391 [M+Na]^+^.

*Methyl 6-deoxy-6-(2-(5-trifluoromethyl-2(1*H*)-pyridone-1-yl-methylene)-anilino)-α-D-glucopyranoside* (**19a**). Compound **19** (135 mg, 0.5 mmol) and **8** (190 mg, 0.6 mmol) were used to prepare compound **19a** (147 mg, 66.2%) as a colorless syrup; [α]^25^_D_ −57 (c 0.10, MeOH); ^1^H-NMR (MeOH-d_4_): δ 7.90 (s, 1H), 7.55 (dd, *J*_1_ = 1.6 Hz, *J*_2_ =7.6 Hz, 1H), 7.15 (m, 2H), 6.70 (d, *J* = 8.0 Hz, 1H), 6.61 (m, 2H), 5.06 (m, 2H, CH_2_), 4.51 (d, *J* = 3.6 Hz, 1H, H-1), 3.67–3.59 (m, 1H), 3.52–3.47 (m, 2H), 3.37–3.31 (m, 1H), 3.22 (s, 1H), 3.16–3.10 (m, 2H), 3.02 (s, 3H, OCH_3_); ^13^C-NMR (MeOH-d_4_): δ 162.9 (C=O), 146.6, 137.0, 137.0, 135.6, 131.0, 130.0, 127.3, 120.3, 119.5, 116.6, 111.1, 110.6, 99.8 (C-1), 73.7, 72.2, 72.1 and 69.0 (C-2, C-3, C-4, C-5), 54.0 (OCH_3_), 48.2 (CH_2_), 44.2 (C-6); HRMS: Calcd for C_20_H_23_N_2_O_6_F_3_: 445.1587 [M+H]^+^, found: 445.1586 [M+H]^+^.

*Methyl* *6-deoxy-6-(4-(5-trifluoromethyl-2(1*H*)-pyridone-1-yl)-anilino)-α-D-mannopyranoside* (**15b**). Compound **15 **(127 mg, 0.5 mmol) and **12** (190 mg, 0.6 mmol) were used to prepare compound **15b** (140 mg, 65.1%) as a colorless syrup; [α]^25^_D_ +82 (c 0.10, MeOH); ^1^H-NMR (MeOH-d_4_): δ 7.98 (s, 1H), 7.67 (dd, *J*_1_ = 2.4 Hz, *J*_2_ = 9.6 Hz, 1H), 7.08 (d, *J* = 8.8 Hz, 2H), 6.76 (d, *J* = 8.4 Hz, 2H), 6.65 (d, *J* = 9.2 Hz, 1H), 4.61 (d, *J*= 1.2 Hz, 1H, H-1), 3.76 (s, 1H), 3.64–3.59 (m, 3H), 3.54–3.51 (m, 1H), 3.32–3.28 (m, 1H), 3.26 (s, 3H, OCH_3_), ^13^C-NMR (MeOH-d_4_): δ 164.5 (C=O), 151.0, 140.6, 140.5, 140.5, 137.3, 130.2, 128.1, 126.2, 123.6, 122.2, 113.0, 111.5, 111.1, 102.8(C-1), 72.6, 72.1, 72.1 and 69.9 (C-2, C-3, C-4, C-5), 55.2 (OCH_3_), 45.5 (C-6); HRMS: Calcd for C_19_H_21_N_2_O_6_F_3_: 431.1431 [M+H]^+^, found: 431.1425 [M+H]^+^.

*Methyl 6-deoxy-6-(3-chloro-4-(5-trifluoromethyl-2(1*H*)-pyridone-1-yl)-anilino)-α-D-mannopyranoside* (**16b**). Compound **16** (145 mg, 0.5 mmol) and **12** (190 mg, 0.6 mmol) were used to prepare compound **16b** (145 mg, 62.5%) as a colorless syrup; [α]^25^_D_ −59 (c 0.10, MeOH); ^1^H-NMR (MeOH-d_4_): δ 7.93 (s, 1H), 7.71 (dd, *J*_1_ = 2.4 Hz, *J*_2_ = 9.6 Hz, 1H), 7.09 (d, *J* = 2.2 Hz, 2H), 6.84 (dd, *J*_1_ = 0.4 Hz, *J*_2_ = 1.6 Hz, 1H), 6.71–6.66 (m, 2H), 4.60 (d, *J* = 1.6 Hz, 1H, H-1), 3.76 (s, 1H), 3.64–3.57 (m, 3H), 3.52–3.48 (m, 1H), 3.30–3.26 (m, 2H), 3.28 (s, 3H, OCH_3_); ^13^C-NMR (MeOH-d_4_): δ 162.7 (C=O), 151.1, 139.8, 139.7, 136.4, 131.3, 128.8, 125.3, 124.7, 122.1, 121.1, 112.3, 111.4, 111.4, 110.2, 109.9, 101.4 (C-1), 71.2, 70.9, 70.7 and 68.4 (C-2, C-3, C-4, C-5), 53.9 (OCH_3_), 43.8 (C-6); HRMS: Calcd for C_19_H_21_N_2_O_6_F_3_Cl: 465.1041 [M+H]^+^, found: 465.1039 [M+H]^+^.

*Methyl* *6-deoxy-6-(3-(5-trifluoromethyl-2(1*H*)-pyridone-1-yl-methylene)-anilino)-α-D-mannopyranoside* (**17b**). Compound **17 **(135 mg, 0.5 mmol) and **12** (190 mg, 0.6 mmol) were used to prepare compound **17b** (154 mg, 69.4%) as a colorless syrup; [α]^25^_D_ −83 (c 0.10, MeOH); ^1^H-NMR (MeOH-d_4_): δ 8.08 (s, 1H), 7.60 (dd, *J*_1_ = 2.8 Hz, *J*_2_ = 9.6 Hz, 1H), 7.06 (dd, *J*_1_ = 7.6 Hz, *J*_2_ = 8.0 Hz, 1H), 6.63–6.60 (m, 3H), 6.52 (d, *J* = 7.2 Hz, 1H), 5.07 (s, 2H, CH_2_), 4.57 (d, *J* = 1.6 Hz, 1H, H-1), 3.74 (t, 1H), 3.62–3.56 (m, 3H), 3.49–3.46 (m, 1H), 3.27–3.25 (m, 1H), 3.17 (s, 3H, OCH_3_); ^13^C-NMR (MeOH-d_4_): δ 162.7 (C=O), 149.3, 138.1, 138.1, 136.5,135.7, 129.3, 124.8, 120.4, 116.0, 112.7, 112.3, 110.2, 109.9, 101.3 (C-1), 71.2, 70.7, 70.3 and 68.6 (C-2, C-3, C-4, C-5), 53.7 (OCH_3_), 52.4 (CH_2_), 44.2 (C-6); HRMS: Calcd for C_20_H_23_N_2_O_6_F_3_: 445.1587 [M+H]^+^, found: 445.1585 [M+H]^+^.

*Methyl* *6-deoxy-6-(4-(5-trifluoromethyl-2(1*H*)-pyridone-1-yl-methylene)-anilino)-α-D-mannopyranoside* (**18b**). Compound **18** (135 mg, 0.5 mmol) and **12** (190 mg, 0.6 mmol) were used to prepare compound **18b** (150 mg, 67.6%) as a colorless syrup; [α]^25^_D_ −67 (c 0.10, MeOH); ^1^H-NMR (MeOH-d_4_): δ 8.06 (s, 1H), 7.58 (dd, *J*_1_ = 2.8 Hz, *J*_2_ = 2.4 Hz, 1H), 7.11 (d, *J* = 8.4 Hz, 2H), 6.65 (d, *J* = 8.4 Hz, 2H), 6.58 (d, *J* = 9.6 Hz, 1H), 5.01 (s, 2H, CH_2_), 4.57 (d, *J*= 1.6 Hz, 1H, H-1), 3.73 (t, 1H), 3.61–3.55 (m, 3H), 3.51–3.46 (m, 1H), 3.27–3.25 (m, 3H), 3.19 (s, 3H, OCH_3_); ^13^C-NMR (MeOH-d_4_): δ 164.2 (C=O), 150.4, 139.1, 139.1, 136.9,130.7, 126.2, 124.9, 123.5, 121.7, 114.4, 111.6, 111.2, 102.7 (C-1), 72.6, 72.1, 71.8 and 70.1 (C-2, C-3, C-4, C-5), 55.1 (OCH_3_), 53.5 (CH_2_), 45.7 (C-6); HRMS: Calcd for C_20_H_23_N_2_O_6_F_3_: 445.1587 [M+H]^+^, found: 445.1589 [M+H]^+^.

*Methyl* *6-deoxy-6-(2-(5-trifluoromethyl-2(1*H*)-pyridone-1-yl-methylene)-anilino)-α-D-mannopyranoside* (**19b**). Compound **19** (135 mg, 0.5 mmol) and **12** (190 mg, 0.6 mmol) were used to prepare compound **19b** (151 mg, 68.0%) as a colorless syrup; [α]^25^_D_ −72 (c 0.10, MeOH); ^1^H-NMR (MeOH-d_4_): δ 7.95 (s, 1H), 7.58 (d, *J* = 9.6 Hz 1H), 7.23–7.17 (m, 2H), 6.72–6.70 (m, 2H), 6.65 (t, *J* = 7.6 Hz, 1H), 5.19–5.09 (m, 2H, CH_2_), 4.62 (s, 1H, H-1), 3.75 (s, 1H), 3.69–3.60 (m, 3H), 3.46–3.42 (m, 1H), 3.34–3.27 (m, 2H), 3.14 (s, 3H, OCH_3_); ^13^C-NMR (MeOH-d_4_): δ 163.0 (C=O), 146.7, 137.1, 137.0, 136.9, 135.7, 130.9, 130.1, 124.6, 121.9, 120.2, 119.5, 116.5, 110.9, 110.9, 110.6, 101.5(C-1), 71.3, 71.0, 70.0 and 68.5 (C-2, C-3, C-4, C-5), 53.8 (OCH_3_), 43.8 (C-6); HRMS: Calcd for C_20_H_23_N_2_O_6_F_3_: 445.1587 [M+H]^+^, found: 445.1581 [M+H]^+^.

### 3.4. General Procedure for the Synthesis of Compounds **15c–19c**

Compound **14a–e** (0.2 mmol) was added to a solution of 0.5 M HCl/MeOH (5 mL) under stirring and under nitrogen atmosphere at room temperature. The progress of reaction was monitored by TLC (6:1CHCl_3_-MeOH). After completion of the reaction the solvent was evaporated under reduced pressure, the residue was purified by flash chromatography (7:1CHCl_3_-MeOH).

*Methyl 6-deoxy-6-(4-(5-trifluoromethyl-2(1*H*)-pyridone-1-yl)-anilino)-β-D-galactofuranoside* (**15c**). Compound **14a** (100 mg, 0.2 mmol) was used to prepare compound **15c** (45 mg, 52.3%) as a colorless syrup; [α]^25^_D_ −48 (c 0.10, MeOH); ^1^H-NMR (MeOH-d_4_): δ 7.93 (s, 1H), 7.62 (dd, *J*_1_ = 2.8 Hz, *J*_2_ = 9.6 Hz, 1H), 7.02 (d, *J* = 8.4 Hz, 2H), 6.66 (d, *J* = 8.8 Hz, 2H), 6.58 (d, *J* = 9.6 Hz, 1H), 4.69 (s, 1H, H-1), 3.85–3.77 (m, 4H), 3.34–3.13 (m, 5H); ^13^C-NMR (MeOH-d_4_): δ 163.3 (C=O), 149.7, 139.4, 136.1, 129.0, 127.0, 121.0, 114.8, 112.4, 110.3, 109.9, 109.3 (C-1), 83.9, 82.0, 77.6 and 68.8 (C-2, C-3, C-4, C-5), 54.3 (OCH_3_), 46.3 (C-6); HRMS: Calcd for C_19_H_21_N_2_O_6_F_3_: 431.1424 [M+H]^+^, found: 431.1420 [M+H]^+^.

*Methyl 6-deoxy-6-(3-chloro-4-(5-trifluoromethyl-2(1*H*)-pyridone-1-yl)-anilino)-β-D-galactofuranoside* (**16c**). Compound **14b** (106 mg, 0.2 mmol) was used to prepare compound **16c** (42 mg, 45.2%) as a yellow syrup; [α]^25^_D_ −64 (c 0.10, MeOH); ^1^H-NMR (MeOH-d_4_): δ 7.95 (s, 1H), 7.69 (dd, *J*_1_ = 2.8 Hz, *J*_2_ = 9.6 Hz, 1H), 7.07 (d, *J* = 8.8 Hz, 1H), 6.79 (t, *J* = 2.4 Hz, 1H), 6.66–6.63 (m, 2H), 4.73 (d, *J* = 1.6 Hz, 1H, H-1), 3.96–3.95 (m, 1H), 3.87–3.85 (m, 2H), 3.83–3.78 (m, 1H), 3.38–3.35 (m, 1H), 3.32 (s, 3H, OCH_3_)，3.25–3.23 (m, 1H), 3.22–3.16 (m, 1H); ^13^C-NMR (MeOH-d_4_): δ 162.7 (C=O), 150.9, 139.7, 136.4, 131.3, 128.9, 125.2, 112.0, 111.2, 109.1, 83.6, 81.7, 77.3 and 68.5 (C-2, C-3, C-4, C-5), 54.0 (OCH_3_), 45.8 (C-6); HRMS: Calcd for C_19_H_20_N_2_O_6_F_3_Cl: 465.1035 [M+H]^ +^, found: 465.1038 [M+H]^+^.

*Methyl* *6-deoxy-6-(3-(5-trifluoromethyl-2(1*H*)-pyridone-1-yl-methylene)-anilino)-β-D-galactofuranoside* (**17c**). Compound **14c** (100 mg, 0.2 mmol) was used to prepare compound **17c** (52 mg, 58.4%) as a yellow syrup; [α]^25^_D_ −34 (c 0.10, MeOH); ^1^H-NMR (MeOH-d_4_): δ 8.07 (s, 1H), 7.58 (dd, *J*_1_ = 2.8 Hz, *J*_2_ = 9.6 Hz, 1H), 7.05–7.01 (m, 1H), 6.60–6.55 (m, 3H), 6.48 (d, *J* = 7.6 Hz, 1H), 5.04 (s, 2H, CH_2_), 4.71 (d, *J*= 1.2 Hz, 1H, H-1), 3.95–3.93 (m, 1H), 3.87–3.83 (m, 2H), 3.80–3.79 (m, 1H), 3.30 (s, 3H, OCH_3_), 3.24 (m, 1H), 3.15–3.10 (m, 1H); ^13^C-NMR (MeOH-d_4_): δ 164.1(C=O), 150.7, 139.5, 138.0, 137.1, 130.7, 121.8, 117.2, 113.6, 113.4, 111.7, 110.5, 85.3, 83.2, 78.9 and 70.0 (C-2, C-3, C-4, C-5), 55.3 (OCH_3_), 53.8 (CH_2_), 47.7 (C-6); HRMS: Calcd for C_20_H_23_N_2_O_6_F_3_: 445.1581 [M+H]^+^, found: 445.1587 [M+H]^+^.

*Methyl 6-deoxy-6-(4-(5-trifluoromethyl-2(1*H*)-pyridone-1-yl-methylene)-anilino)-β-D-galactofuranoside* (**18c**). Compound **14d** (100 mg, 0.2 mmol) was used to prepare compound **18c** (47 mg, 52.8%) as a yellow syrup; [α]^25^_D_ −75 (c 0.10, MeOH); ^1^H-NMR (MeOH-d_4_): δ 8.06 (s, 1H), 7.56 (dd, *J*_1_ = 2.8 Hz, *J*_2_ = 9.6 Hz, 1H), 7.09 (d, *J* = 8.4 Hz, 2H), 6.61–6.54 (m, 3H), 4.98 (s, 2H, CH_2_), 4.70 (d, *J* = 2.0 Hz, 1H, H-1), 3.94–3.91 (m, 1H), 3.85–3.82 (m, 2H), 3.80–3.76 (m, 1H), 3.29 (s, 3H, OCH_3_)，3.25–3.23 (m, 3H), 3.16–3.11 (m, 1H); ^13^C-NMR (MeOH-d_4_): δ 162.8 (C=O), 148.9, 137.8, 135.5, 129.4, 123.4, 120.3, 112.6, 109.1, 83.7, 81.9, 77.4 and 68.6 (C-2, C-3, C-4, C-5), 54.0 (OCH_3_), 52.1 (CH_2_), 46.2 (C-6); HRMS: Calcd for C_20_H_23_N_2_O_6_F_3_: 467.1400 [M+Na]^+^, found: 465.1380 [M+Na]^+^.

*Methyl* *6-deoxy-6-(2-(5-trifluoromethyl-2(1H)-pyridone-1-yl-methylene)-anilino)-β-D-galactofuranoside* (**19c**). Compound **14e** (100 mg, 0.2 mmol) was used to prepare compound **19c** (49 mg, 55.1%) as a yellow syrup; [α]^25^_D_ −72 (c 0.10, MeOH); ^1^H-NMR (MeOH-d_4_): δ 7.97 (s, 1H), 7.57 (dd, *J*_1_ = 2.8 Hz, *J*_2_ = 7.2 Hz, 1H), 7.16–7.10 (m, 2H), 6.68 (d, *J* = 8.0 Hz, 1H), 6.63–6.59 (m, 2H), 5.09 (s, 2H, CH_2_), 4.73 (s, 1H, H-1), 3.97–3.96 (m, 1H), 3.88–3.84 (m, 3H), 3.36–3.17 (m, 5H); ^13^C-NMR (MeOH-d_4_): δ 163.0 (C=O), 146.5, 137.3, 137.2, 135.7, 130.4, 129.9, 120.3, 119.5, 116.5, 110.8, 109.1, 84.0, 81.8, 77.5 and 68.5 (C-2, C-3, C-4, C-5), 54.0 (OCH_3_), 48.5 (CH_2_), 46.2 (C-6); HRMS: Calcd for C_20_H_23_N_2_O_6_F_3_: 445.1581 [M+H]^+^, found: 445.1583 [M+H]^+^.

## 4. Conclusions

In conclusion, we report the design, synthesis and biological evaluation of some carbohydrate-modified 1-(substituted aryl)-5-trifluoromethyl-2(1*H*) pyridones. Our studies suggest that some modifications of pirfenidone analogues with carbohydrates appear to increase the inhibitory activity against NIH 3T3 cell proliferation. Among the compounds tested, compound **19a**, which was synthesized by modification of pirfenidone analogue **19** with glucose, demonstrated the highest cell-based inhibitory activity (IC_50_ = 0.17 mM).
